# YAP1 controls the N-cadherin-mediated tumor-stroma interaction in melanoma progression

**DOI:** 10.21203/rs.3.rs-2944243/v3

**Published:** 2023-07-28

**Authors:** Yao Xio, Lilni Zhou, Thomas Andl, Yuhang Zhang

**Affiliations:** University of Cincinnati; University of Cincinnati; University of Cincinnati

**Keywords:** Yes-associated protein 1, N-cadherin, fibroblasts, melanoma, microenvironment

## Abstract

Epithelial-to-mesenchymal transition (EMT) is crucial for melanoma cells to escape keratinocyte control, invade underlying dermal tissues, and metastasize to distant organs. The hallmark of EMT is the switch from epithelial cadherin (E-cadherin) to neural cadherin (N-cadherin), allowing melanoma cells to form a homotypic N-cadherin-mediated adhesion with stromal fibroblasts. However, how “cadherin switching” is initiated, maintained, and regulated in melanoma remains unknown. Here, we show that upon Yes-associated protein 1 (YAP1) ablation in cancer-associated fibroblasts (CAFs), the progression of a BRAF-mutant mouse melanoma was significantly suppressed *in vivo*, and overexpressing YAP1 in CAFs accelerated melanoma growth. CAFs require the YAP1 function to proliferate, migrate, remodel the cytoskeletal machinery and matrix, and promote cancer cell invasion. By RNA-Seq, N-cadherin was identified as a major downstream effector of YAP1 signaling in CAFs. YAP1 silencing led to N-cadherin downregulation in CAFs, which subsequently induced the downregulation of N-cadherin in neighboring melanoma cells. N-cadherin downregulation inhibited the PI3K-AKT signaling pathway in melanoma cells and suppressed melanoma growth *in vivo*, supporting the role of N-cadherin as an adhesive and signaling molecule in melanoma cells. This finding suggests that YAP1 depletion in CAFs induces the downregulation of p-AKT signaling in melanoma cells through the N-cadherin-mediated interaction between melanoma cells and CAFs. Importantly, our data underscore that CAFs can regulate N-cadherin-mediated interactions with melanoma cells. Thus, disentangling cadherin-mediated cell–cell interactions can potentially disrupt tumor-stroma interactions and reverse the tumor cell invasive phenotype.

## Introduction

Despite the advancements and renewed hopes inspired by novel targeted and immune therapies, there is still a long way to go to find a cure for advanced melanoma. A hallmark of metastatic tumors is their ability to escape primary sites, invade surrounding tissues, and migrate to distant sites [1, 2]. The malignant phenotype is achieved by cancer cells at the invasive front to break the surrounding extracellular matrix (ECM) barrier through reciprocal tumor-stroma interactions [3]. During this process, melanoma cells develop a dynamic and mutually supportive relationship with neighboring cells and the matrix in the host microenvironment, which consists of a heterogeneous mix of noncancer cells, including endothelial cells, immune cells, and fibroblasts. Genetically stable fibroblasts that surround and infiltrate melanoma stroma, often termed CAFs, remodel the ECM and secrete chemical factors, which together constitute the backbone of a protective “paradise” that allows melanoma cells to grow, migrate and resist therapeutic agents [4]. Conceivably, targeting CAFs could potentially abrogate microenvironment-mediated resistance and improve treatment outcomes in metastatic melanoma.

YAP1 is an oncoprotein and a downstream transcriptional coactivator in the Hippo signaling pathway [5]. YAP1 regulation by the highly conserved Hippo signaling pathway involves a cascade of protein serine kinases, including mammalian sterile twenty-like kinases 1/2 (MST1/2, Hippo homologs; also known as STK3/K4) and large tumor suppressor kinases 1/2 (LATS1/2, Warts homologs) [6]. When Hippo signaling is active, YAP1 is phosphorylated by LATS1/2 for cytoplasmic retention, ubiquitination, and degradation. When Hippo signaling is off, YAP1 remains unphosphorylated, translocates to the nucleus, and drives the transcription of target genes. The Hippo signaling pathway is considered an important tumor-suppressor pathway, and its dysregulation has been noted in a variety of human cancers, in which YAP1 nuclear translocation abnormally activates target gene expression and enables cancerous cells to overcome contact inhibition, grow, and spread uncontrollably [7].

YAP1 activity is believed to be important for fibroblast differentiation, activation, and proliferation [8–10]. Lee *et al*. reported that YAP1 expression is highly increased in dermal fibroblasts in cutaneous wounds, and YAP1 silencing significantly delayed the wound-healing process [11]. CAFs in solid tumors have characteristics similar to those of myofibroblasts [12]. Calvo *et al*. reported that nuclear YAP1 activity is required to maintain CAF tumor-promoting phenotypes, including matrix stiffening, cancer cell invasion and angiogenesis [13]. It is believed that reciprocal interactions between YAP1 activity in CAFs and tissue stiffness and mechanical stress in the tumor microenvironment drive a continuous process of maintaining CAF phenotypes and malignancy [14].

Previously, we showed that YAP1 is highly expressed in the nuclei of CAFs in human melanoma, and the biological functions of stromal fibroblasts, especially their ECM-remodeling ability, are inhibited when YAP1 expression is silenced [10]. However, the molecular mechanisms underlying the functional connection between YAP1 activity and the tumor-promoting functions of CAFs have not been fully identified. In this study, we established two mouse models that carry either YAP1-deficient CAFs or YAP1-overexpressing CAFs and demonstrated that YAP1 is important for CAFs to promote *in vivo* melanoma growth. YAP1-deficient CAFs exhibit significant cytoskeletal changes with reduced levels of actin stress fibers, focal adhesions, and myosin. We identified N-cadherin as a YAP1 downstream effector in CAFs. N-cadherin is a major molecule that mediates strong cell–cell adhesion and promotes cell viability and movement. Surprisingly, we found that N-cadherin downregulation in CAFs induced the downregulation of N-cadherin in melanoma cells, subsequently causing inhibited p-AKT signaling and cell proliferation. Taken together, our work uncovers a novel YAP1-regulated cellular mechanism that controls melanoma cell phenotypes and proliferation through the N-cadherin-mediated interaction between melanoma cells and CAFs.

## Materials and methods

### Cell lines

The murine D4M melanoma cell line was purchased from Kerafast (Boston, MA) [15]. The human melanoma cell lines A375 and SK-MEL-24 were purchased from the American Type Culture Collection (ATCC, Manassas, VA) and maintained in Dulbecco’s modified Eagle medium (DMEM) in a humidified incubator at 37°C with 5% CO2. Two human CAF cell lines (224350P1/M50 and DT01027P1/M27) were isolated from surgically excised human melanoma tissues and obtained from Asterand bioscience (Detroit, MI). All culture media were supplemented with 10% (v/v) fetal bovine serum (FBS) and 1% (v/v) 10,000 U/ml penicillin and 10,000 U/ml streptomycin. All cell culture reagents were purchased from Thermo Fisher Scientific (Rochester, NY) unless otherwise stated. The isolation and maintenance of primary human fibroblasts were approved by the Institutional Review Board and the Institutional Biosafety Office of the University of Cincinnati. Experimental procedures involving biosafety issues were carried out under the University of Cincinnati Institutional Biosafety Committee protocol 16-08-17-01.

### Mice

The *α-SMA-CreER*^*T2*^ mouse strain was generated and authorized by Dr. Pierre Chambon at The Institute of Genetics and Molecular and Cellular Biology (IGBMC) in France and kindly provided by Dr. Richard T. Premont at Duke University. The *Yap1*^*loxP/loxP*^ mouse strain was generated and authorized by Dr. Eric Olson at UT Southwestern and kindly provided by Dr. Mei Xin at the Cincinnati Children’s Hospital Medical Center. C57BL/6J (B6), *Rosa-rtTA* (Jax 005670) and *tetO-Yap1* (Jax 031279) mouse strains were obtained from the Jackson Laboratory (Bar Harbor, ME, USA). Both mouse strains were backcrossed to the B6 background. *Yap1*^*loxP/loxP*^ mice were crossed with *α-SMA-CreER*^*T2*^ mice for several generations to generate *Yap1*^*loxP/loxP*^; *α-SMA-CreER*^*T2*^ mice. *tetO-Yap1*, *α-SMA-CreER*^*T2*^ and *Rosa-rtTA* mouse strains were crossed to generate triple *tetO-Yap1*;*α-SMA-CreER*^*T2*^; *Rosa-rtTA* mice. Mice were genotyped by polymerase chain reaction (PCR) analysis of genomic DNA extracted from tail biopsies. The *Yap1*^*loxP/loxP*^ alleles were genotyped using the following primer pair: forward: 5′ ACATGTAGGTCTGATGCCAGAGGAGG 3′ and reverse: 5′ AGGCTGAGACAGGGGATCTCTGTGAG 3′. The *tetO-Yap1* alleles were genotyped using the following primer pair: forward: 5′ GCTGCCACCAAGCTAGATAAAG 3′ and reverse: 5′ CTGAACTTGTGGCCGTTTAC 3′. The *Rosa-rtTA* alleles were genotyped with the following primer pair: forward: 5′ AAGACCGCGAAGAGTTTGTC 3′ and reverse: 5′ AAAGTCGCTCTGAGTTGTTAT 3′. The presence of the Cre transgene (*α-SMA-CreER*^*T2*^) was identified by PCR using the following primer pair: forward: 5′ CGGTCTGGCAGTAAAAACTAT 3′ and reverse: 5′ CAGGGTGTTATAAGCAATCCC 3′. All mice were housed in the Laboratory Animal Services Facility of the University of Cincinnati under an artificial 12 h/12 h light-dark cycle and were allowed free access to food and water. The Institutional Animal Care and Use Committee of the University of Cincinnati (IACUC) approved all experimental procedures involving mice (22-08-19-01).

### Mouse melanoma induction

**Figure 1:**
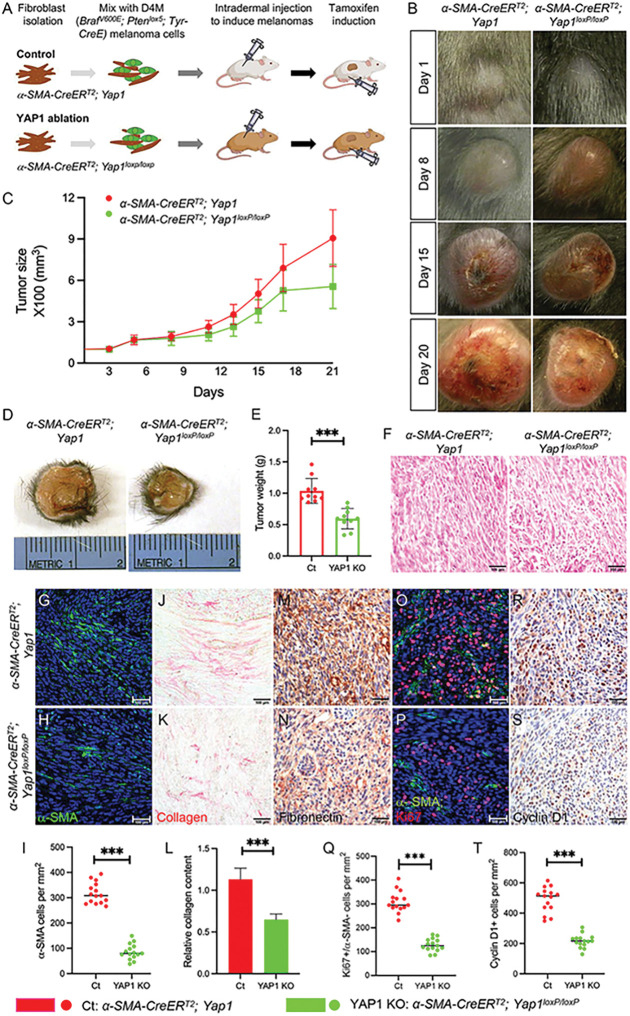
*Braf*^*V600E*^*; Pten*^*lox5/lox5*^ melanoma growth is suppressed by YAP1 deficiency in CAFs. A. Illustration of the tumor grafting mouse model for studying the *in vivo* effects of YAP1 ablation in CAFs on melanoma growth. D4M melanoma cells (green) mixed with uninduced control Fb (*a-SMA-CreER*^*T2*^*; Yap1*) or mutant Fb (*a-SMA-CreER*^*T2*^*; Yap1*^*loxp/loxp*^) were injected intradermally into the flanks of mice carrying the same genotypes as fibroblasts. Tumors were allowed to grow to a volume of approximately 62.5 cubic millimeters when tamoxifen was administered to induce YAP1 ablation in mutant *Yap1*^*loxp/loxp*^ CAFs and numbered day 1. The growth of tumors from both the control *Braf*^*V600E*^*; Pten*^*lox5/lox5*^*-a-SMA-CreER*^*T2*^*; Yap1* (Ct) and mutant *Braf*^*V600E*^*; Pten*^*lox5/lox5*^*-a-SMA-CreER*^*T2*^*; Yap1*^*loxp/loxp*^ (YAP1 KO) groups was monitored and compared. n=10. B. Representative pictures of Ct melanoma (left panel) and YAP1 KO melanoma (right panel) on days 1, 8, 15, and 20. C. Tumor sizes were measured and compared between Ct melanomas and YAP1 KO melanomas. n=10. D. Representative pictures of Ct and YAP1 KO melanomas at day 21. E. Tumor weight comparison between Ct and YAP1 KO melanomas. The graph shows the tumor weight comparison between the two groups on day 21. Each data point represents the weight of one tumor on day 21. n=10. The box indicates the average tumor weight, and error bars indicate the mean ± SD. F. Histological sections of Ct melanoma and YAP1 KO melanoma were photographed under a light microscope. G-H. Images show a-SMA staining of CAFs in Ct and YAP1 KO melanomas. The nuclei were counterstained using DAPI (blue). I. Graph shows the numbers of a-SMA+ fibroblasts per mm^2^ counted in Ct and YAP1 KO melanomas. Fifteen random fields from three melanoma pairs were counted. n=15. J-K. Images show collagen staining of melanoma tissue sections in the indicated groups. L. Quantitative comparison of collagen contents in Ct and YAP1 KO melanomas by collagen extraction and colorimetric measurement. n=4. M-N. Images show ECM protein fibronectin expression in Ct and YAP1 KO melanomas. O-P. Images show a-SMA and Ki67 staining of melanoma tissue sections in the indicated groups. Q. Graph shows the comparison of the numbers of a-SMA^−^ cells that are Ki67+ in each group. Each data point represents the number of a-SMA^−^; Ki67+ cells per mm^2^ counted in each melanoma. R-S. Representative images of IHC cyclin D1 staining of melanoma tissue sections in the indicated groups. T. Graph shows the numbers of cyclin D1+ cells in Ct and YAP1 KO melanomas. Each data point represents the number of cyclin D1+ cells per mm^2^ counted in one melanoma. For all staining pictures, the scale bar represents 100 μm. In all statistical graphs, data are represented as the mean ± SD. *, P£0.05; **, P£0.01; ***, P£0.001; ns, not significant.

To generate melanomas carrying YAP1-deficient CAFs in mice, as shown in Fig. 1A, fibroblasts of genotype *α-SMA-CreER*^*T2*^; *Yap1*^*loxP/loxP*^ and control *α-SMA-CreER*^*T2*^; *Yap1* were isolated from 2- or 3-day-old neonatal littermates without induction and validated by immunostaining for the expression of α-SMA, vimentin, S100A4, keratin 14, and TRP1. Subsequently, a mix of 2 × 10^5^ D4M melanoma cells and uninduced *α-SMA-CreER*^*T2*^; *Yap1*^*loxP/loxP*^ or control *α-SMA-CreER*^*T2*^; *Yap1* fibroblasts at a ratio of 1:1 was injected intradermally into the flanks of mice possessing the same genotype as injected fibroblasts. Mice were monitored daily for tumor formation. To induce the activation of Cre recombinase and *Yap1* knockout in *α-SMA-CreER*^*T2*^; *Yap1*^*loxP/loxP*^ fibroblasts, all mice underwent intraperitoneal injection of 10 mg/ml tamoxifen (Sigma–Aldrich, St. Louis, MO) in corn oil at 1 mg/g body weight for seven consecutive days after tumors reached a volume of approximately 62.5 cubic millimeters (counted as day 1). Meanwhile, the size of the tumors was measured and recorded for three weeks. Mice were euthanized when the tumor size exceeded 20% of body size, and tumors were harvested for various analyses.

**Figure 2:**
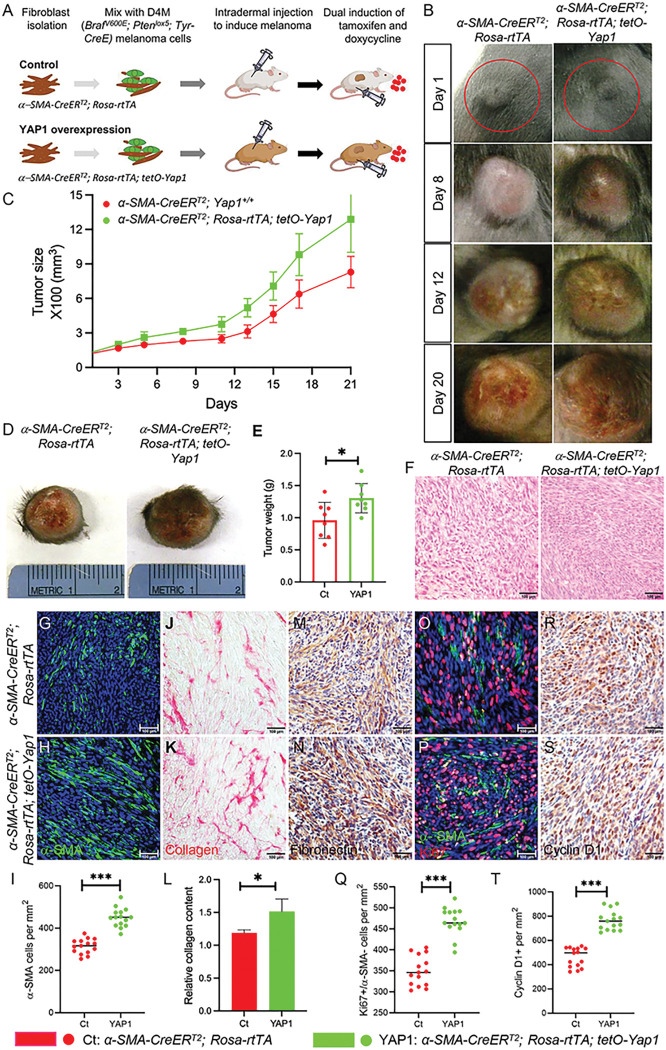
YAP1 overexpression in CAFs accelerates *Braf*^*V600E*^*; Pten*^*lox5/lox5*^ melanoma progression. A. Illustration of the tumor grafting mouse model for studying the *in vivo* effects of YAP1 overexpression in CAFs on melanoma progression. D4M melanoma cells (green) were mixed with either uninduced control Fb (*a-SMA-CreER*^*T2*^*; Rosa-rtTA*) or mutant Fb (*a-SMA-CreER*^*T2*^*; Rosa-rtTA; tetO-Yap1*) and then injected intradermally into the flanks of mice carrying the same genotypes as fibroblasts. Tumors were allowed to grow to a volume of approximately 62.5 cubic millimeters before tamoxifen was administered, and mice were fed with a Dox diet (small red dots) to induce YAP1 overexpression in mutant CAFs. The growth of tumors from both control *Braf*^*V600E*^*; Pten*^*lox5/lox5*^*-a-SMA-CreER*^*T2*^*; Rosa-rtTA* (Ct) and mutant *Braf*^*V600E*^*; Pten*^*lox5/lox5*^*-a-SMA-CreER*^*T2*^*; Rosa-rtTA; tetO-Yap1* groups (YAP1) was closely monitored and compared. n=8. B. Representative pictures of Ct melanomas and YAP1 melanomas on days 1, 8, 12, and 20. C. Tumor size was measured and compared between Ct melanomas and YAP1 melanomas. n=8. D. Representative pictures of Ct melanoma and YAP1 melanoma on day 21. E. Tumor weight comparison between the Ct and YAP1 groups. The graph shows the tumor weight comparison between the two groups on day 21. Each data point represents the weight of one tumor on day 21. n=8. The box indicates the average tumor weight, and error bars indicate the mean ± SD. F. Histological sections of Ct melanoma and YAP1 melanoma were photographed under a light microscope. G-H. Images show a-SMA staining of CAFs in Ct and YAP1 melanomas. The nuclei were counterstained blue using DAPI. I. Graph shows the numbers of a-SMA+ fibroblasts per mm^2^ counted in Ct and YAP1 melanomas. n=15. J-K. Images show collagen staining of melanoma tissue sections in the indicated groups (red). L. Quantitative comparison of collagen contents in Ct and YAP1 melanomas by collagen extraction and colorimetric measurement. n=3. M-N. Images show ECM protein fibronectin expression in Ct and YAP1 melanomas. O-P. Images show a-SMA and Ki67 staining of melanoma tissue sections in the indicated groups. Q. Graph shows the numbers of a-SMA^−^ melanoma cells that are Ki67+ in each group. Each data point represents the number of a-SMA^−^; Ki67^+^ cells per mm^2^ counted in each melanoma. R-S. Representative images of IHC cyclin D1 staining of melanoma tissue sections in the indicated groups. T. Graph shows the numbers of cyclin D+ cells in Ct and YAP1 melanomas. Each data point represents the number of cyclin D+ cells per mm^2^ counted in each melanoma. For all staining pictures, the scale bar represents 100 μm. In all statistical graphs, data are represented as the mean ± SD. *, P£0.05; **, P£0.01; ***, P£0.001; ns, not significant.

To generate melanomas carrying YAP1-overexpressing CAFs in mice, as shown in Fig. 2A, fibroblasts of genotype *α-SMA-CreER*^*T2*^; *Rosa-rtTA; tetO-Yap1* and control *α-SMA-CreER*^*T2*^; *Rosa-rtTA* were isolated from 2- or 3-day-old neonatal littermates without induction and validated by immunostaining for the expression of α-SMA, vimentin, S100A4, keratin 14, and TRP1. Subsequently, a mix of 1 × 10^5^ D4M melanoma cells and 1 × 10^5^ uninduced *α-SMA-CreER*^*T2*^; *Rosa-rtTA; tetO-Yap1* or control *α-SMA-CreER*^*T2*^; *Rosa-rtTA* fibroblasts was injected intradermally into the flanks of mice carrying the same genotype as injected fibroblasts. Mice were monitored daily for tumor formation. To induce the activation of Cre recombinase and YAP1 overexpression in *α-SMA-CreER*^*T2*^; *Rosa-rtTA; tetO-Yap1* fibroblasts, all mice were fed a doxycycline (Dox) diet and underwent intraperitoneal injection of 10 mg/ml tamoxifen (Sigma–Aldrich, St. Louis, MO) in corn oil at 1 mg/g body weight for seven consecutive days after tumors reached a volume of approximately 62.5 cubic millimeters (counted as day 1). Meanwhile, the size of the tumors was measured and recorded for three weeks. Mice were euthanized when the tumor size exceeded 20% of body size, and tumors were harvested for various analyses.

### Melanoma cell isolation

To isolate melanoma cells from mouse tumors, we labeled D4M melanoma cells with red fluorescent protein (RFP) for easy identification and isolation. Mouse melanomas carrying YAP1-deficient CAFs were generated as described above. Fresh melanomas were excised from mice on day 21. After removing necrotic and connective tissues, the remaining tissues were minced using a scalpel blade into small pieces and incubated with 5 ml of 2.5 mg/ml collagenase IV at 37°C with stirring for one hour to generate a single-cell suspension. After digestion, the mixture was filtered through a 40 μm cell strainer to remove tissue debris and cell clumps. The filtered cell suspension was centrifuged at 1500 rpm for 10 minutes. Afterwards, cell pellet was washed with DMEM medium with 10% FBS twice and then suspended in PBS solution with 2% FBS for fluorescence-activated cell sorting (FACS) to isolate RFP-tagged D4M cells.

### Histology, immunofluorescence staining, and immunohistochemistry

Tumor tissues were fixed in 10% formalin overnight at 4°C and embedded in paraffin. Five-micron-thick paraffin-embedded tumor tissue sections were prepared for hematoxylin and eosin (H&E) staining and immunostaining as described previously [16, 17]. For histological analysis, paraffin sections were stained using a standard H&E staining protocol. Slides were mounted using VectaMount permanent mounting medium (Vector lab, Burlingame, CA) and analyzed using a brightfield microscope.

For immunostaining, the following primary antibodies were used: anti-α-SMA (Invitrogen, A2547, 1:200), anti-fibronectin (Sigma–Aldrich, F3648, 1:200), anti-Ki67 (BD Pharmingen, #550609, 1:50), anti-Cyclin D1 (Cell Signaling, #2978, 1:50), anti-N-cadherin (Abcam, ab18203, 1:100), anti-p-Akt (phospho S473, Abcam, ab66138, 1:100), anti-YAP1 (Proteintech, 13584-1-AP, 1:500), and anti-TE7 (Millipore, CBL271, 1:200). After incubation with primary antibodies, slides were washed with PBS three times, incubated with the corresponding biotin-conjugated secondary antibodies at room temperature for one hour, and then with either fluorochrome-conjugated streptavidin for immunofluorescence or VECTASTAIN Elite ABC Reagents (Vector Laboratories, Burlingame, CA) for immunohistochemistry. Nuclei were counterstained with DAPI (blue) for immunofluorescence or hematoxylin (blue) for immunohistochemistry. Images were taken using a Nikon Eclipse 80i fluorescence microscope. The number of positively stained cells in each high-power field (40X) was counted using the particle analysis function of ImageJ software (NIH). The number of positive cells per square millimeter was calculated by multiplying the number of cells counted in each field by 4.5.

### Collagen staining

The collagen content in melanoma tissues was evaluated using the Picrosirius Red staining kit (American MasterTech, Lodi, CA) according to the manufacturer’s instructions. After staining, the slides were washed with 1% acetic acid for one minute, dehydrated in 100% ethanol, cleared using xylene, and mounted using VectaMount permanent mounting medium.

### Quantification of collagen content

To quantify collagen content in melanoma tissues, a Sirius Red/Fast Green Collagen Staining Kit (Chondrex, Redmond, WA) was used according to the manufacturer’s instructions. Briefly, after the slide was incubated with the dye solution at room temperature for 30 minutes, the dye solution was removed, and the slide was rinsed with distilled water until the water became colorless. One milliliter of dye extraction buffer was added to each slide to elute the dye from dyed tissues. A 200 μl dye extraction solution from each slide was collected for absorbance measurements at 540 nm and 605 nm using a microplate reader. The collagen content in each sample was normalized to the total protein content.

### Generation of inducible YAP1-deficient CAFs

To ablate YAP1 expression in CAFs, M27 and M50 were transduced with doxycycline-dependent inducible lentivirus expressing shRNAs that specifically target YAP1 expression (V3SH7669-225152043, V3SH7669-225222498, V3SH7669-226435710 from GE Dharmacon, Lafayette, CO). A nontargeting shRNA lentivirus was used to generate control M27 and M50. The inducible lentiviral shRNA vector, which uses the Tet-On inducible system, only allows the expression of either YAP1-targeting shRNA or nontargeting shRNA when cells are treated with doxycycline. The expression of GFP is driven by the same tetO promoter so that the transduction and shRNA expression upon doxycycline treatment can be visually tracked by green fluorescence. Briefly, CAFs were seeded in 6-well tissue culture plates. When the cells reached 50% confluence, lentiviral particles were added and incubated with the cells for 16 hours. The medium containing viral particles was then replaced with fresh DMEM supplemented with 0.5% FBS. To select transduced fibroblasts, medium containing 10 μg/ml puromycin was added and maintained for three days. To assess whether puromycin selection was complete, 500 ng/ml doxycycline (Fisher Scientific, Pittsburgh, PA) was added for 72 hours to induce the expression of shRNAs and GFP. The efficiency of inhibiting YAP1 expression by shRNA was determined by Western blotting and qPCR.

### N-cadherin siRNA silencing

Silencer siRNAs targeting N-cadherin (siRNA ID: s2771) and scramble Silencer^®^ siRNA control were purchased from Thermo Fisher Scientific (Rochester, NY). Melanoma cells and CAFs were seeded in 6 cm dishes at an initial cell density of 1 × 10^5^ cells, cultured for 24 hours until 60–70% confluence, and then transfected with siRNA using Lipofectamine RNAiMAX (Thermo Fisher Scientific, Rochester, NY). According to the manufacturer’s protocol, N-cadherin siRNA (10 μM) was diluted in 250 μl of Opti-MEM I reduced serum medium (Thermo Fisher Scientific, Rochester, NY) and mixed with 15 μl of Lipofectamine RNAiMAX in 250 μl of Opti-MEM I Reduced Serum Medium. After incubation at room temperature for 10 minutes, the mixture was added to the cells and incubated for three days. Afterward, the medium containing siRNA and RNAiMAX was replaced with regular DMEM culture medium.

### Human melanoma cell labeling

The human melanoma cell lines A375 and SK-MEL-24 were transduced with a nontargeting control hEF1α-TurboRFP lentivirus expressing red fluorescent protein (RFP) (GE Dharmacon, cat# VSC6573). Lentivirus transduction was carried out as described above. RFP expression and transduction efficiency were confirmed by flow cytometry.

### Chamber slide immunofluorescence staining

1.5 × 10^4^ cells were counted and seeded in one well of an 8-well Nunc^™^ Lab-Tek^™^ II chamber slide (Thermo Fisher Scientific, Rochester, NY). The cells were fixed in 4% paraformaldehyde for 10 minutes at room temperature and then permeabilized using 0.05% Triton-100 for 10 minutes on ice for immunofluorescence staining. After permeabilization, the cells were washed three times with PBS and blocked in 10% normal goat serum for 1 hour at room temperature. Primary antibodies recognizing F-actin (Thermo Fisher, R415, 1:60), paxillin (BD Biosciences, 610051, 1:200), fibronectin (Sigma, F3648, 1:200), α-SMA (Thermo Fisher, 14-9760-82, 1:200), S100A4 (Thermo Fisher, 16105-1-AP, 1:200), N-cadherin (Abcam, ab18203, 1:100), MYH10 (Cell Signaling Technology, 3404, 1:100), and MLC2 (Cell Signaling Technology, 3672, 1:100) were then added to each specific chamber and incubated overnight at 4°C. The next day, after washing with PBS three times, an Alexa Fluor 488- or 594-conjugated secondary antibody (Thermo Fisher Scientific, Rochester, NY) was added to the corresponding well for a one-hour incubation at room temperature. The slides were mounted with VECTASHIELD Antifade Mounting Medium (Vector Laboratories, Burlingame, CA) and coverslipped. Images were acquired using a Nikon Eclipse 80i fluorescence microscope. For EdU staining, a Click-iT^™^ Plus EdU Cell Proliferation Kit was used for imaging according to the manufacturer’s instructions. The number of EdU + cells and total cell number in each high-power field (40X) were counted using the particle analysis function of ImageJ software. The TUNEL assay was performed using an In Situ Cell Death Detection Kit following the standard protocol provided by the manufacturer (Sigma, St. Louis, MO).

### Cell viability and proliferation assays

For cell number counting, 1 × 10^5^ cells were seeded in one 6 cm dish and cultured for seven days. The cells were collected on days 1, 3, 5 and 7 for cell number counting using a hemocytometer. At least three repeats were performed for each indicated cell line per assay, and cell number counting was performed a minimum of three times. The MTT assay was performed as we previously published [17].

### Generation of melanoma cell-conditioned culture medium

To prepare melanoma cell-conditioned culture medium, A375 or SK-MEL-24 melanoma cells were seeded in 10 cm dishes. After the cells reached confluency, the medium was replaced with serum-free medium for another 24-hour culture. Afterwards, the medium was collected and centrifuged at 1000 × g for 10 minutes to remove cell debris for future use.

### Transwell migration assay

To perform the transwell migration assay, 3.5 × 10^5^ CAFs were seeded on the insert with a permeable membrane (Greiner Bio-One, Kremsmünster, Austria) in a 6-well plate. To assess the response and migratory ability of CAFs to different chemoattractants with and without YAP1 expression, melanoma cell-conditioned medium was added to the bottom well or melanoma cells were seeded in the well, ensuring that CAFs on the membrane were in contact with the medium. After 48 hours, CAFs that migrated through the membrane were stained and imaged. Because CAFs were transduced with GFP, migrated CAFs on the opposite side of the insert were first captured using a Cytation 1 cell imaging multimode reader and then fixed with paraformaldehyde for crystal violet staining. The migration area was defined as the area occupied by green fluorescent CAFs and was quantified using ImageJ.

### Collagen gel contraction assay

A collagen gel contraction assay was performed as reported previously [10, 16, 17]. After 72 hours of doxycycline induction, 1 × 10^5^ CAF cells were resuspended in 500 μl of 1 mg/ml collagen solution and transferred into one well of a 24-well tissue culture plate. After a 30-minute incubation in a humidified incubator at 37°C, one ml of fresh medium was added on top of the gel for a 72-hour incubation. Afterwards, gels were detached from the wall of each well and allowed to contract as indicated. Pictures of the gels were taken using a Nikon digital camera every 24 hours, and ZEN 2.3 software was used to measure the diameters. The gel contraction percentage was calculated by dividing the difference in gel diameters between 0 hours and 72 hours by the diameter at 0 hours.

### Confocal reflection microscopy (CRM)

CRM was performed as reported previously [10, 16, 17]. After a 72-hour incubation, collagen fiber distribution was assessed using a Zeiss LSM 710 confocal microscope at 40X magnification. Images were acquired at a depth of at least 100 mm inside the gel to avoid edge effects. Images of at least ten areas of each gel were randomly captured for 3D reconstruction of the matrix using ImageJ. Fiber connectivity was calculated using ImageJ with the BoneJ plugin (http://bonej.org).

### 3D invasion assay

Collagen gels were prepared as described in the gel contraction assay. 100 μl of gel mixture was placed on the transwell insert in a 24-well plate and allowed to solidify for 30 minutes. Afterwards, 5 × 10^4^ CAFs were resuspended in 300 μl of gel mixture and added to the top of the solidified gel, and 1 ml of medium was added for incubation overnight. After 24 hours, the medium was removed, and 5 × 10^4^ A375 melanoma cells suspended in 100 μl medium were added on top of collagen gel embedded with CAFs. To allow melanoma cells to invade into the underlying gel, gel assemblies carrying CAFs and A375 melanoma cells in 24-well plates were placed in an incubator for up to 15 days. On days 10 and 15, collagen gels were removed and fixed with 4% paraformaldehyde overnight at 4°C. Fixed gels were collected for paraffin section preparation and histological analysis to determine the distance of melanoma cell invasion in the matrices. The collagen content was determined as described above. The distance of melanoma cell invasion was measured using ImageJ.

### Quantitative real-time PCR assay

RNA was extracted from cultured cells and melanoma tissues using a PurelinkTM RNA Mini kit (Thermo Fisher, Waltham, MA) according to the manufacturer’s protocol. For 3D coculture spheroids, RNA was extracted using an RNeasy Plus Micro Kit (Qiagen, Hilden, Germany). Briefly, frozen tumor tissues were added to an RNase-free mortar containing liquid nitrogen and ground thoroughly into powder using an RNase-free pestle before transferring to an RNase-free microcentrifuge tube. Based on the weight of the powder, enough lysis buffer was added for the subsequent homogenization and RNA extraction steps. For cultured cells and spheroids, samples were collected and washed once using ice-cold PBS for RNA extraction. RNA concentration was determined using a NanoDrop spectrophotometer. cDNAs were generated by reverse transcription using a SuperScript IV first-strand synthesis system (Thermo Fisher, Waltham, MA). qPCRs were performed using SYPR green master mix power on a StepOnePlus real-time PCR system (Applied Biosystems, Waltham, MA). qPCR primers for fibronectin, tenascin C, talin, and N-cadherin were purchased from realtimeprimers.com (Philadelphia, PA). The relative expression level of each gene was normalized to the level of GAPDH. The data shown were generated from at least three independent repeats.

### 3D spheroid cell coculture

Cocultured cell spheroids were formed by mixing 5000 RFP-tagged melanoma cells with 5000 CAFs in a total volume of 100 μl in 96-well plates (Thermo Fisher Scientific, Rochester, NY) with a low cell-adhesion surface. Fluorescent images of spheroids were taken every 24 hours for up to 72 hours using a Cytation 1 cell imaging multimode reader [10]. The RFP area and intensity were recorded each time. To count the RFP + melanoma cell number in the spheroids, 12 spheroids from each group were collected and digested using 2 mg/ml collagenase IV (Thermo Fisher Scientific, MA) for 30 minutes at 37°C with stirring to generate a single cell suspension. A Countess II Automated Cell Counter (Thermo Fisher Scientific, Rochester, NY) was used to quantify the RFP + melanoma cell number. The average melanoma cell number in each spheroid was calculated by dividing the total RFP + melanoma cell number by 12. For immunofluorescence staining, spheroids were washed once with cold PBS, fixed in 4% paraformaldehyde, and dehydrated using ethanol. After dehydration, spheroids were embedded in 1% agarose gel for paraffin section preparation and immunostaining.

### RNA-Seq

Total RNA was extracted from GFP/M50 and YAP1-GFP/M50 cells after a 3-day doxycycline induction using a PureLink^™^ RNA Mini Kit. RNA-Seq was performed as single-end sequencing at the University of Cincinnati Genomics, Epigenomics and Sequencing core using the Illumina NextSeq 550 system. Generated fastq files were validated, processed, and analyzed using the UCSC human hg19 reference genome on the A.I.R. platform (Sequentia Biotech, Barcelona, Spain). Differentially expressed genes were identified using the DESeq2 package [18]. Genes with an adjusted p value < 0.05 and a fold change > or < 2 were considered significant. Heatmaps of differentially expressed genes were generated using A.I.R. A volcano plot to visualize significant gene expression was generated using the OmicStudio tools [19]. Gene Ontology (GO) functional enrichment analysis was performed using A.I.R. Kyoto Encyclopedia of Genes and Genomes (KEGG) pathway enrichment analysis was performed using DAVID Bioinformatics Resources 6.8 [20]. The most affected sets of genes in YAP1-GFP/M50 upon YAP1 ablation were determined using the GSEA software package 4.3.1 [21].

### Western Blotting

Standard Western blotting was performed as previously reported [10, 16, 17]. The following antibodies were used: anti-YAP1 (Proteintech, 13584-1-AP, 1:2500), anti-N-cadherin (Abcam, ab18203, 1:500), anti-Akt (Cell Signaling, #2920S, 1:1000), anti-p-Akt (S473) (Cell Signaling, #4060S, 1:1000), anti-MYH9 (Cell Signaling, 3403, 1:1000), anti-MYH10 (Cell Signaling, 8824, 1:1000), anti-MLC2 (Cell Signaling, 3672, 1:1000), anti-b-actin (Thermo Fisher, MA5-15739, 1:3000), anti-b-tubulin (Thermo Fisher, MA5-16308, 1:3000), and anti-GAPDH (Thermo Fisher, 437000, 1:1000). For chemiluminescent Western blotting using X-ray film, the blots were incubated with either HRP-conjugated goat anti-mouse IgG (H + L) antibody (Cell Signaling, #7076, 1:2000) or HRP-conjugated goat anti-rabbit IgG (H + L) antibody (Cell Signaling, #7074, 1:1000) at room temperature for one hour. Afterwards, the membranes were washed and incubated with ECL Western blotting detection substrate (GE Healthcare, Chicago, IL). for five minutes. The blots were then exposed to X-ray film. For fluorescent Western blotting using the Odyssey imaging systems, membranes were incubated with either IRDye 680RD goat anti-rabbit IgG secondary antibody (LI-COR, 926-68071, 1:2000), IRDye 800CW donkey anti-rabbit IgG secondary antibody (LI-COR, 926-32213, 1:2000), IRDye 680RD donkey anti-mouse IgG secondary antibody (LI-COR, 926-68072, 1:2000), or IRDye 800CW goat anti-mouse IgG secondary antibody (LI-COR, 926-32210, 1:2000) at room temperature for one hour. The membranes were then washed again using TBST and processed for scanning, visualization and quantification using the Odyssey CLx imaging system (LI-COR, model #9140).

### Statistical analysis

All quantitative results were obtained from a minimum of three independent experiments. Data were analyzed using the GraphPad Prism 9 software package (GraphPad Software Inc., San Diego, CA) and expressed as the mean ± SD. The mean difference was determined by Student’s t tests and considered statistically significant at P < 0.05.

## Results

### Stromal YAP1 signaling contributes to melanoma progression in vivo

To assess the contribution of YAP1 signaling in CAFs to BRAF-mutant melanoma progression *in vivo*, we generated a transgenic mouse model, *α-SMA-CreER*^*T2*^; *Yap1*^*loxP/loxP*^, which allows the inducible ablation of YAP1 expression in CAFs [22, 23]. In the *α-SMA-CreER*^*T2*^ transgene, the expression of sequestered Cre recombinase is driven by a *α-SMA* promoter [24], which is known to be a major gene expressed in CAFs [25]. *Yap1*^*loxP/loxP*^ harbors homozygous loxP-flanked *Yap1* alleles, which can be recombined into *Yap1*-null alleles by activated Cre recombinase [23]. To induce melanomas, isolated fibroblasts of genotype either *α-SMA-CreER*^*T2*^; *Yap1*^*loxP/loxP*^ (YAP1 group) or *α-SMA-CreER*^*T2*^; *Yap1* (control group) were mixed with oncogenic D4M melanoma cells, which carry the *Braf*^*V600E*^ activating mutation and are PTEN-deficient, and injected intradermally into the flanks of recipient mice of the same genotype as CAFs (Fig. 1A). When the tumors reached an approximate size of 62.5 mm^3^, the mice were administered tamoxifen by intraperitoneal injection to activate sequestered Cre recombinase and knock out the *Yap1* gene in CAFs (Fig. S1A-B).

As shown in Fig. 1B-C, *Braf*^*V600E*^; *Pten*^*lox5/lox5*^ melanomas carrying YAP1-deficient CAFs (named YAP1 KO) grew slower than tumors carrying CAFs expressing normal levels of YAP1 (named Ct). Twenty-one days after tamoxifen injection when mice were sacrificed, YAP1 KO melanomas were significantly smaller in size (Fig. 1D) and lighter in weight (Fig. 1E) (555.91 ± 160.70 mm^3^, 0.59 ± 0.16 g) than Ct tumors (906.43 ± 205.25 mm3, 1.04 ± 0.20 g), suggesting that *Braf*^*V600E*^; *Pten*^*lox5/lox5*^ melanoma growth was suppressed upon the loss of YAP1 in CAFs. H&E staining revealed that the structure of YAP1 KO melanomas was less compact than that of Ct tumors with more intercellular spaces (Fig. 1F). The number of α-SMA-positive (α-SMA+) CAFs was reduced in melanomas upon YAP1 ablation (Fig. 1G-I). CAFs are the major producers of collagen and ECM proteins in the tumor stroma. As shown in Fig. 1J-K and M-N, the levels of collagen fibers and fibronectin were significantly reduced in YAP1 KO melanomas. Quantification of collagen contents further confirmed the reduction in collagen production in melanomas carrying YAP1-deficient CAFs (Fig. 1L). Consistent with the reduced YAP1 KO tumor size and weight, the number of melanoma cells (a-SMA-negative, α-SMA-) that were Ki67-positive (Ki67+) and the number of Cyclin D1-positive (Cyclin D1+) cells were both decreased in YAP1 KO melanomas (Fig. 1O-T). The data suggest that YAP1 activity is important for CAFs to support melanoma cell proliferation *in vivo*.

### YAP1 overexpression in CAFs accelerates tumor progression

Next, we addressed the impact of upregulating YAP1 signaling in CAFs on melanoma progression. As shown in Fig. 2A, we generated a triple transgenic mouse strain, *α-SMA-CreER*^*T2*^; *Rosa-rtTA; tetO-Yap1*, that inducibly overexpresses YAP1 protein in α-SMA + fibroblasts and determined whether YAP1-overexpressing α-SMA + fibroblasts could accelerate melanoma progression. In the *tetO-Yap1* transgene, the expression of YAP1 is controlled by a doxycycline-inducible tetO promoter [26] and requires the presence of reverse tet transactivator (rtTA) and doxycycline. The expression of rtTA by the *Rosa-rtTA* transgene is prevented by a loxP-flanked STOP cassette [27]. Thus, the generation of rtTA can only occur when active Cre recombinase is present to remove the STOP cassette, which is provided by the *α-SMA-CreER*^*T2*^ transgene when tamoxifen is injected, leading to the overexpression of YAP1 protein in α-SMA + cells (Fig. S1C-D).

Interestingly, we observed that melanomas carrying YAP1-overexpressing fibroblasts (named YAP1) grew more quickly than control tumors carrying wild-type fibroblasts (Fig. 2B-C). On day 21, when the tumors were collected, the average size and weight of YAP1 melanomas (1287.99 ± 286.13 mm^3^, 1.30 ± 0.23 g) exceeded those of control tumors carrying wild-type fibroblasts (829.98 ± 136.48 mm^3^, 0.96 ± 0.28 g) (Fig. 2D-E). Histological analysis showed that YAP1 melanomas had a more compact internal structure than control tumors (Fig. 2F). The number of α-SMA + CAFs per mm^2^ was increased from 314 ± 35 in control melanomas to 450 ± 44 in YAP1 melanomas (Fig. 2G-I). The contents of collagen (Fig. 2J-L) and fibronectin (Fig. 2M-N) were also higher in YAP1 melanomas than in control melanomas. In contrast to the YAP1 KO mouse model, as shown in Fig. 2O-Q, overexpressing YAP1 in CAFs increased the number of Ki67 + melanoma cells per mm^2^ to 468 ± 33, while control melanomas had an average of 350 ± 35. Similarly, the number of Cyclin D1 + melanoma cells was also increased in YAP1 melanomas (Fig. 2R-T). The data are consistent with increased tumor size and weight upon YAP1 overexpression in CAFs and further confirm that YAP1 indeed plays a crucial role in CAFs to support melanoma growth.

### YAP1 regulates the biological properties of CAFs

**Figure 3:**
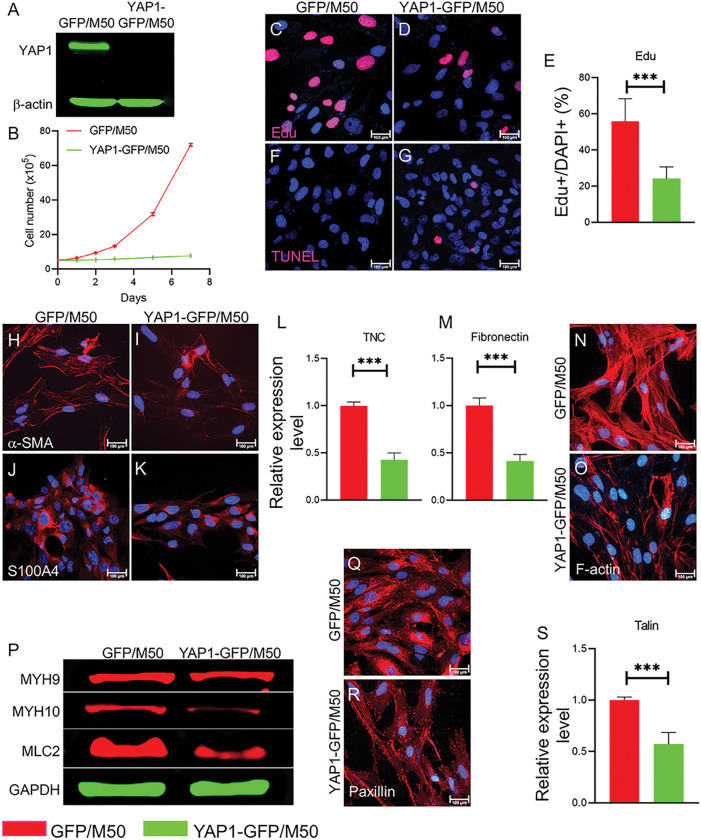
YAP1 is essential for the biological properties of CAFs. All cells were treated with 500 ng/ml doxycycline for 72 hours for experiments unless otherwise stated. A. Western blot showing YAP1 expression in cultured GFP/M50 and YAP1-GFP/M50 cells. B. The numbers of GFP/M50 and YAP1-GFP/M50 in culture medium were counted at days 0, 1, 2, 3, 5, and 7 for comparison. n=3. C-D. Images show EdU staining of GFP/M50 and YAP1-GFP/M50 with DAPI nuclear counterstaining. E. Graph shows the statistical comparison of the percentages of EdU+ cells in cultured GFP/M50 and YAP1-GFP/M50 cells from three independent experiments. F-G. Images show TUNEL+ cells in cultured GFP/M50 and YAP1-GFP/M50 cells by TUNEL assay. H-K. Images show a-SMA and S100A4 staining of GFP/M50 and YAP1-GFP/M50. L-M. The expression of the ECM proteins TNC and fibronectin in GFP/M50 or YAP1-GFP/M50 cells was quantified by qPCR. GAPDH was used as an internal control. n=3 N-O. Images show F-actin staining of GFP/M50 and YAP1-GFP/M50 with DAPI counterstaining. P. Western blot showing the expression of MYH9, MYH10 and MLC2 in GFP/M50 and YAP1-GFP/M50 cells. Q-R. Images show paxillin staining of GFP/M50 and YAP1-GFP/M50 with DAPI counterstaining. S. The expression of talin in GFP/M50 and YAP1-GFP/M50 cells was quantified by qPCR. GAPDH was used as an internal control. n=3. The expression levels of the indicated proteins were normalized to the expression level of GAPDH. For all staining pictures, the scale bar represents 100 μm. In all statistical graphs, data are represented as the mean ± SD. *, P£0.05; **, P£0.01; ***, P£0.001; ns, not significant.

To further evaluate the mechanism that controls CAF phenotypes after YAP1 loss, we established inducible YAP1-deficient CAFs using shRNA in different human melanoma-derived CAF lines, including M27 and M50 [17]. Three different YAP1-targeting shRNAs (Fig. S2A-D) were evaluated for their efficiencies in silencing YAP1 expression. YAP1-GFP/Fb-3 shRNA (V3IHSHEG-6373360) was found to have the highest inhibitory efficiency and was selected for subsequent experiments (Fig. S2E), although both siRNAs showed significant effects in reducing cell viability (Fig. S2F). CAFs transduced with shRNA (V3IHSHEG-6373360) were named YAP1-GFP/M50 and YAP1-GFP/M27. Nontargeting shRNA-transduced M27 and M50 were used as the controls and named GFP/M50 and GFP/M27, respectively. YAP1 depletion in M27 and M50 was confirmed by Western blotting and qPCR (Fig. 3A and S2G-H). The loss of YAP1 expression suppressed the proliferation of M50 and M27 cells (Fig. 3B and S3A) but did not cause increased cell apoptosis, which was confirmed by EdU staining and TUNEL assay (Fig. 3C-G and S3B-C). There was no clear change in the morphology of YAP1-deficient M27 and M50 cells. The expression of CAF markers, including α-SMA and S100A4 [25], was strongly decreased in YAP1-deficient CAFs, YAP1-GFP/M50 (Fig. 3H-K) and YAP1-GFP/M27 (Fig. S3F-I). Quantification analysis showed reduced fibronectin and Tenascin C (TNC) contents in YAP1-deficient CAFs (Fig. 3L-M and Fig. S3D-E).

CAFs exhibit remodeled cytoskeletal structure with increased intracellular tension. As shown in Fig. 3N-P and S3J-O, YAP1 deficiency led to reduced levels of stress fiber F-actin, myosin heavy chain MYH10, and myosin light chain MLC2 in CAFs. In addition, the expression of the focal adhesion proteins paxillin and talin, which are involved in cytoskeletal regulation, was suppressed (Fig. 3Q-S and S3P-Q). The data suggest that YAP1 function is involved in the control of cytoskeletal machinery.

### CAFs require YAP1 to maintain matrix remodeling ability

**Figure 4:**
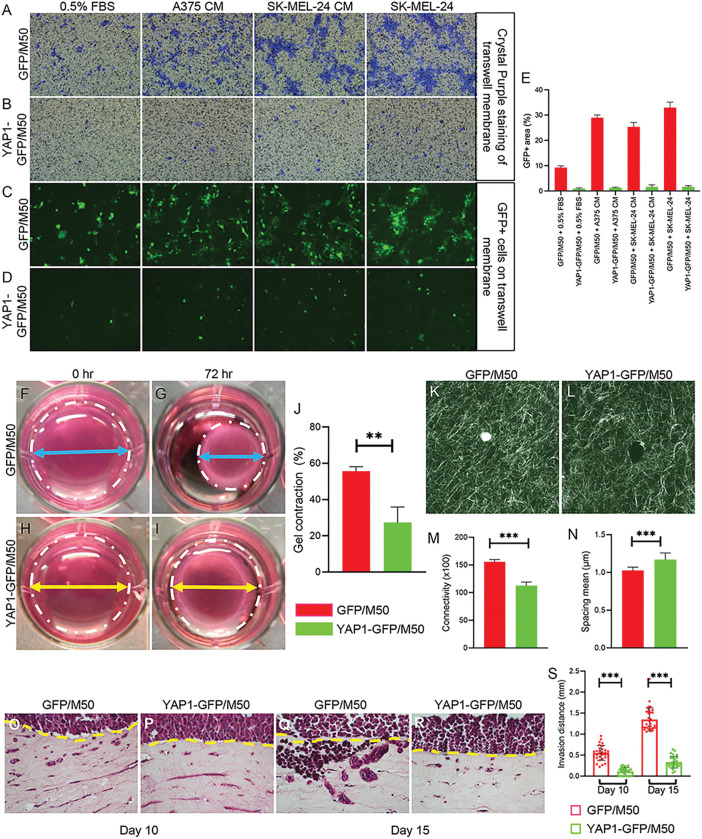
YAP1 regulates the migratory and ECM remodeling abilities of CAFs. All cells were treated with 500 ng/ml doxycycline for 72 hours for experiments unless otherwise stated. A-D. Images show the migratory response of GFP/M50 and YAP1-GFP/M50 to the indicated culture conditions at 48 hours in the transwell migration assay by crystal violet staining (A-B) and green fluorescence imaging (C-D). E. Bar graph shows the percentages of the area occupied by green fluorescent GFP/M50 and YAP1-GFP/M50 on the Transwell membranes at the 48th hour. n=6. F-I Representative images of collagen gel contraction assays using GFP/M50 and YAP1-GFP/M50 at 0 hours and 72 hours. The gel in each well is circled by a white dashed line. The blue (GFP/M50) and yellow (YAP1-GFP/M50) arrow lines indicate the diameter of the contracted gels. J. Statistical quantification of the relative percentages of gel contraction by GFP/M50 and YAP1-GFP/M50 from 0 hours to 72 hours. n=3. K-L. CRM images of gels embedded with GFP/M50 and YAP1-GFP/M50 at 72 hours. M-N. CRM images were analyzed using ImageJ with the BoneJ plugin to compare connectivity and spacing between the gels embedded with GPF/M50 and YAP1-GFP/M50. O-R. Representative images of melanoma cell migration in collagen gels embedded with GFP/M50 and YAP1-GFP/M50 after 10 days and 15 days. n=3. The melanoma front is indicated by a yellow line. S. Statistical quantification of the migrating distances by melanoma cells in the gels embedded by GFP/M50 and YAP1-GFP/M50 at day 10 and day 15. In all statistical graphs, data are quantified and shown as the mean ± SD. *, P£0.05; **, P£0.01; ***, P£0.001; ns, not significant.

Migration, matrix contraction, and ECM remodeling require cytoskeletal contraction and remodeling [28, 29]. Since YAP1 deficiency led to reduced expression of cytoskeletal proteins, we assessed the contribution of YAP1 to the migratory ability of CAFs using the transwell migration assay. As shown in Fig. 4A-E and S3R, after 48 hours, when cultured in DMEM with 0.5% FBS, the percentage of migrating GFP-positive (GFP+) YAP1-GFP/M50 cells on the insert membrane was 0.92 ± 0.35%, which was significantly less than that of GFP/M50 (9.25 ± 0.71%). More significantly, the percentages of the GFP + area on the membrane were significantly increased to 28.54 ± 0.74% and 25.73 ± 1.19% when GFP/M50 was cultured in the media conditioned by A375 and SK-MEL-24 CM, respectively. The percentage was further increased to 33.26 ± 2.58% when SK-MEL-24 cells were seeded directly in the bottom well. However, YAP1-GFP/M50 and YAP1-GFP/M27 failed to respond to either conditioned media or direct stimulation in SK-MEL-24 cells cultured in the bottom well.

Gel contraction assays showed that the ability of CAFs to remodel the collagen matrix was reduced after YAP1 depletion (Fig. 4F-J and S3S-V). Next, to visualize and quantify collagen fiber alignment and reorganization, collagen fiber distribution was assessed using confocal reflection microscopy (CRM). The density of collagen fibers in the gel was significantly lower in collagen gel embedded with YAP1-deficient CAFs than in collagen gel embedded with wild-type CAFs (Fig. 4K-L). CRM analysis revealed decreased connectivity and increased fiber spacing in collagen gel embedded with YAP1-deficient CAFs (Fig. 4M-N). The CRM data correlated well with the results shown by the collagen gel contraction assays, highlighting the importance of YAP1 for the ability of CAFs to remodel the ECM.

To understand how the ECM remodeled by CAFs influences the invasion of melanoma cells, we performed a 3D collagen gel invasion assay. GFP/M50 or YAP1-GFP/M50 cells were embedded in collagen gel and seeded onto 24-well transwell inserts. Melanoma cell A375 was added on top of the collagen gel embedded with CAFs and placed in a 24-well plate for culture for up to 15 days. Gel samples were collected on days 10 and 15. Histological staining showed a significant reduction in melanoma cell invasion in collagen gel populated with YAP1-deficient CAFs (Fig. 4O-R). On day 15, the distance of melanoma cells invading in gel embedded with CAFs was four times greater than that of YAP1-deficient CAFs (to 1.34 ± 0.28 mm vs. 0.33 ± 0.14 mm in Fig. 4S), suggesting that CAFs require YAP1 functions to make the gel accessible for A375 to invade.

### N-cadherin is a YAP1 target in CAFs

**Figure 5:**
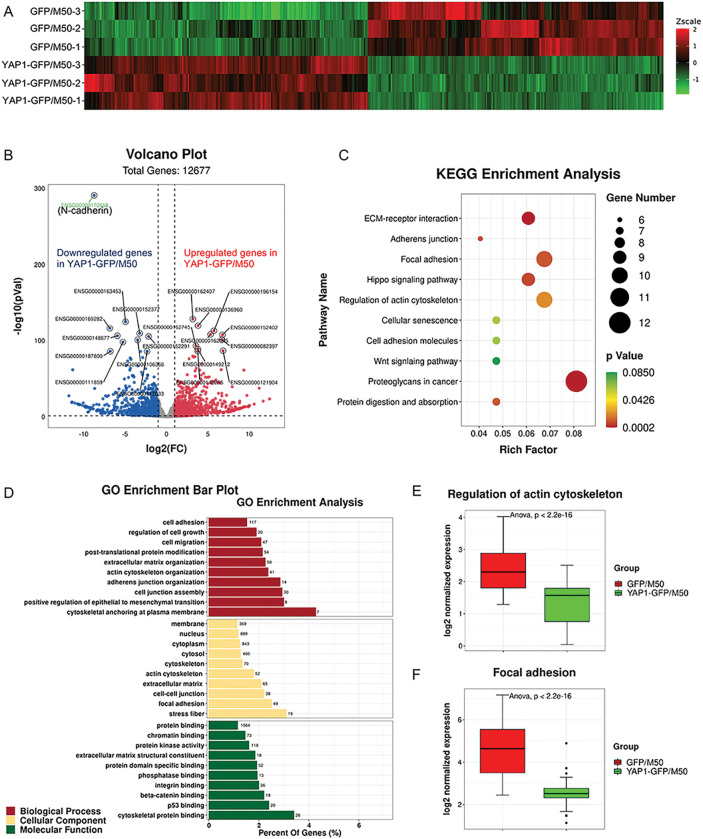
N-cadherin is a YAP1 target in CAFs. A. Heatmap of the expression patterns (Z-scaled FPKM values) of the differentially expressed genes between GFP/M50 and YAP1-GFP/M50. Changes in expression levels are displayed from green (less expressed) to red (more expressed). B. Volcano plot shows the relationship between the fold-change (on the X-axis) and the significance of the differential expression test (Y-axis) for each gene between GFP/M50 and YAP1-GFP/M50. Red dots represent upregulated genes and blue dots represent downregulated genes in YAP1-GFP/M50 comparing to GFP/M50. Gray dots represent the genes that are not significantly differentially expressed between GFP/M50 and YAP1-GFP/M50. C. The scatter plot shows the KEGG enrichment analysis results. The size and color of dots represent gene numbers and p values. The richfactor is the ratio of the number of differentially expressed genes enriched in the indicated pathway to the number of all genes annotated in this pathway. D. The bar plot shows GO enrichment analysis results in three major biological processes using the GO database. E-F. Gene set enrichment analysis (GSEA) demonstrated thatYAP1 has a significant correlation with biological functions related to regulation oftheactin cytoskeleton and focal adhesion.

To obtain a global picture of the underlying mechanisms by which YAP1 regulates the functional properties of CAFs and identify YAP1-regulated genes that are involved in a CAF-elicited melanoma program, we performed RNA-Seq to compare the gene expression profiles between GFP/M50 and YAP1-GFP/M50. As shown in Fig. 5A, a list of differentially expressed genes was generated by high-stringency and comparative analysis of RNA-Seq data using the Air platform (https://transcriptomics.sequentiabiotech.com/). A total of 1147 genes that were upregulated at least twofold and 1140 genes that were downregulated at least twofold in YAP1-GFP/M50 are shown in a volcano plot (Fig. 5B). We are particularly interested in understanding the genes that were downregulated in YAP1-GFP/M50, as they may be relevant for identifying the YAP1-mediated mechanism by which CAFs interact with melanoma cells. Therefore, KEGG pathway analysis and Gene Ontology (GO) enrichment analysis were performed [30–32]. As shown in Fig. 5C, KEGG enrichment analysis of the top 300 downregulated genes revealed that proteoglycans in cancer, regulation of actin cytoskeleton, and focal adhesion are the most affected pathways in CAFs after the loss of YAP1 expression. The most enriched subclasses by GO enrichment analysis of all 1140 downregulated genes were cytoskeletal anchoring at the plasma membrane, stress fiber, and cytoskeletal protein binding (Fig. 5D). Gene set enrichment analysis (GSEA) showed that the expression levels of the genes in the KEGG pathways of regulation of actin cytoskeleton and focal adhesion were indeed suppressed in YAP1-GFP/M50 compared to those of GFP/M50 (Fig. 5E-F). Interestingly, among the downregulated genes, CDH2, the gene encoding N-cadherin, appears to be the most significantly downregulated gene in CAFs upon YAP1 ablation, as shown in the volcano plot (Fig. 5B), and could potentially function as a key downstream effector of YAP1 signaling in CAFs due to its known role in tumor cell-fibroblast adhesion.

### N-cadherin deficiency in CAFs leads to the downregulation of N-cadherin in melanoma cells

**Figure 6:**
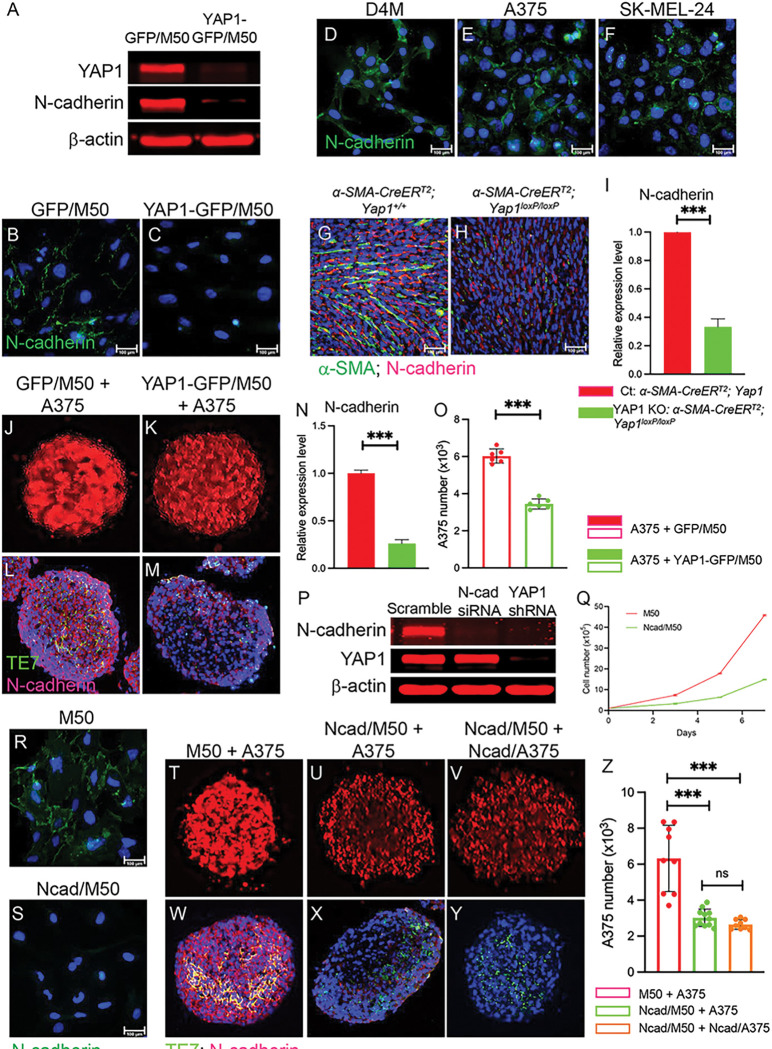
N-cadherin deficiency in CAFs leads to N-cadherin downregulation in melanoma cells. A. Western blot showing the loss of N-cadherin in YAP1-GFP/M50 cells upon YAP1 ablation. B-C; Images show N-cadherin staining of GFP/M50 and YAP1-GFP/M50 with blue DAPI counterstaining. D-F. Images show N-cadherin staining of D4M, A375, and SK-MEL-24 cells with blue DAPI counterstaining. G-H. CAFs and N-cadherin expression in mouse melanoma sections were visualized by coimmunostaining using an anti-a-SMA antibody (green) and anti-N-cadherin antibody (red). I. Quantification of N-cadherin expression in the indicated mouse melanoma tissues by qPCR and normalized to the internal control GAPDH. n=3. J-K. Fluorescence images show RFP-tagged A375 cells in cocultured spheroids as indicated. L-M. Images show TE7 and N-cadherin staining of cocultured spheroids as indicated using an anti-TE7 antibody (green) and an anti-N-cadherin antibody (red). N. Quantitative analysis of N-cadherin expression in spheroids coculturedas indicated. n=3. O. Graph shows the numbers of A375 cells in coculturedspheroids as indicated. n=6. P. Western blot shows that N-cadherin expression was silenced in M50 cells transfected with N-cadherin siRNA with normal YAP1 expression. In contrast, YAP1 ablation in M50 led to N-cadherin downregulation. Scrambled siRNA was used as a control. Q. Comparison of the numbers of N-cadherin siRNA-transfected M50 and scramble siRNA-transfected M50 cells in culture for seven days. n=3. R-S. Images show N-cadherin staining of scramble siRNA-transfected M50 and N-cadherin siRNA-transfected M50 with blue DAPI counterstaining. T-V. Fluorescence images show RFP-tagged A375 cells in cocultured spheroids as indicated. W-Y. Images show TE7 and N-cadherin staining of cocultured spheroids as indicated using an anti-TE7 antibody (green) and an anti-N-cadherin antibody (red). Z. Graph shows the numbers of A375 cells in cocultured spheroids as indicated. n=9. For all staining pictures, the scale bar represents 100 μm. In all statistical graphs, data are represented as themean ± SD. *, P£0.05; **, P£0.01; ***, P£0.001; ns, not significant.

N-cadherin is a transmembrane protein that is known to function in cell–cell adhesion [33]. Importantly, the E-cadherin to N-cadherin switch is known as a major part of the EMT event in tumor progression and metastasis [34]. Western blotting and immunostaining demonstrated the loss of N-cadherin after YAP1 silencing in CAFs (Fig. 6A-C and S4A-C). N-cadherin is expressed in both the mouse melanoma cell line D4M and the human melanoma cell lines A375 and SK-MEL-24 (Fig. 6D-F). We then checked the expression of N-cadherin in our mouse melanoma model (Fig. 1A), in which YAP1 is ablated in CAFs. Surprisingly, as shown in Fig. 6H, the number of CAFs was dramatically decreased in the tumor, as shown by α-SMA staining, and the expression of N-cadherin was markedly reduced, especially in melanoma cells (α-SMA^−^), suggesting that N-cadherin downregulation caused by YAP1 deficiency in CAFs induced N-cadherin downregulation in melanoma cells. To validate this finding, we performed a qPCR analysis of N-cadherin expression using tumor tissues isolated from control and YAP1 KO tumors. As shown in Fig. 6I, the expression of N-cadherin in YAP1 KO tissues was three times lower than that in Ct tissues, indicating that N-cadherin expression was indeed downregulated in melanoma cells upon YAP1 ablation in CAFs. Next, we cocultured A375 cells with either GFP/M50 or YAP1-deficient YAP1-GFP/M50 cells using an *in vitro* 3D coculture system and assessed N-cadherin expression. The spheroids formed by A375 and YAP1-GFP/M50 appeared to have more interspaces and lower levels of N-cadherin expression (Fig. 6K, M) than the spheroids formed by A375 and GFP/M50 (Fig. 6J, L), which was confirmed by qPCR analysis (Fig. 6N). The observation of a loose spheroid structure is consistent with what we observed in mouse melanoma carrying YAP1-deficient CAFs (Fig. 1F). Because A375 was tagged with red fluorescence, the number of A375 cells in each spheroid was counted and compared using a Countess II Automated Cell Counter. As expected, the spheroids formed by A375 and YAP1-deficient M50 contained a lower number of A375 than the ones formed by A375 and M50 (Fig. 6O). The findings were confirmed using M27 and the melanoma cell line SK-MEL-24 (Fig. S4D-I). The reduction in melanoma cells in spheroids also explained a potential reason why the spheroids formed by A375 and YAP1-deficient M50 appeared loose.

Next, to confirm that ablating N-cadherin expression in CAFs does have similar effects as YAP1 depletion and to substantiate our finding that N-cadherin is the major downstream target of YAP1, we used siRNA to silence N-cadherin expression in GFP/M50 cells. After depleting N-cadherin expression, YAP1 was still expressed in CAFs (Fig. 6P, R-S), suggesting that N-cadherin is indeed a downstream effector of YAP1. However, the loss of N-cadherin in M50 suppressed cell proliferation (Fig. 6Q), partially mirroring the effects of YAP1 on CAF proliferation shown in Fig. 3B. We utilized an *in vitro* 3D coculture system to determine whether N-cadherin deficiency in CAFs can lead to a change in N-cadherin in melanoma cells, similar to YAP1 deficiency. As shown in Figs. 6X and S4M-N, depleting N-cadherin in CAFs led to the downregulation of N-cadherin in the melanoma cell lines A375 and SK-MEL-24. Furthermore, the spheroids formed by A375 and N-cadherin-deficient M50 (Fig. 6U), SK-MEL-24 and N-cadherin-deficient M27 (Fig. S4K), and N-cadherin-deficient A375 and N-cadherin-deficient M50 (Fig. 6V) displayed loose structure compared to M50 + A375 spheroids (Fig. 6T) and M27 + SK-MEL-24 spheroids (Fig. S4J). Spheroids were digested using collagenase to make single cell suspensions for cell counting. The number of A375 and SK-MEL-24 cells in the spheroids was significantly reduced after the loss of N-cadherin expression in CAFs but close to the number of A375 cells in the spheroids formed by N-cadherin-deficient A375 and N-cadherin-deficient M50 cells (Fig. 3Z and S4O), suggesting that YAP1 deficiency and N-cadherin deficiency have similar effects in regulating tumor-stroma interactions.

### N-cadherin loss in melanoma cells downregulates p-AKT signaling

**Figure 7:**
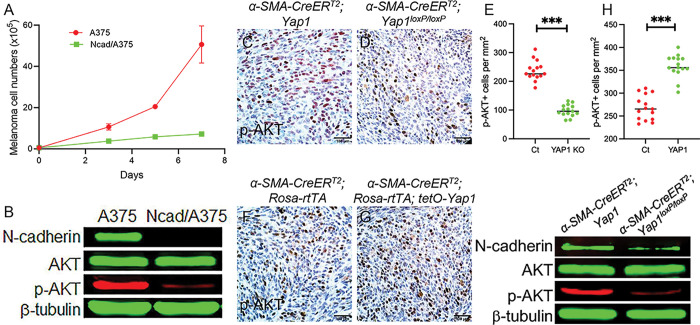
N-cadherin downregulation inhibits PI3K/AKT signaling in BRAF-mutant melanoma cells. A. Comparison of the numbers of N-cadherin siRNA-transfected A375 and scramble siRNA-transfected A375 cells in culture for seven days. n=3. B. Western blot shows N-cadherin, AKT, and p-AKT expression in A375 cells transfected with scramble siRNA or N-cadherin siRNA. C-D. Representative images of p-AKT expression in melanoma tissues containing Ct CAFs and YAP1-deficient CAFs. E. Graph shows the numbers of p-AKT+ cells per mm^2^ counted in Ct and YAP1 KO melanomas. n=15. F-G. Representative images of p-AKT expression in melanoma tissues containing Ct CAFs and YAP1-overexpressing CAFs. H. Graph shows the numbers of p-AKT+ cells per mm^2^ counted in Ct and YAP1 melanomas. n=15. I. Expression of N-cadherin, AKT and p-AKT in melanoma cells isolated from Ct and YAP1 KO melanomas was assessed by Western blotting. b-tubulin was used as an internal control. In all statistical graphs, data are represented as the mean ± SD. *, P£0.05; **, P£0.01; ***, P£0.001; ns, not significant.

It was reported previously that N-cadherin contributes to the proliferation of different cell types via the PI3K/AKT signaling pathway [35]. We examined the proliferation of melanoma cells after N-cadherin ablation and found that N-cadherin depletion in A375 and SK-MEL-24 indeed led to reduced cell proliferation (Fig. 7A and S4P). As shown in Fig. 7B, S4Q, and S5, p-AKT was significantly decreased in N-cadherin-deficient melanoma cells, suggesting that N-cadherin in melanoma cells regulates the activation of the PI3K/AKT signaling pathway. Furthermore, AKT signaling was significantly suppressed in D4M melanoma cells when YAP1 expression was silenced in CAFs in the melanoma stroma (Fig. 7C-E). However, an increased number of p-AKT-positive (p-AKT+) cells was found in the melanomas containing YAP1-overexpressing CAFs (Fig. 7F-H). To confirm that AKT signaling was indeed downregulated in melanoma cells upon YAP1 ablation in CAFs, tumor cells were isolated from melanomas containing YAP1-deficient CAFs for AKT Western blotting. As shown in Fig. 7I, both N-cadherin and p-AKT were reduced in melanoma cells, suggesting that AKT signaling in BRAF-mutant melanoma cells was regulated through the N-cadherin-N-cadherin interaction between melanoma cells and CAFs.

## Discussion

Cutaneous melanoma arises from the malignant transformation of skin melanocytes and is known for its high propensity to metastasize [36]. Formation of a “reactive” tumor stroma is a wound-like reaction of the stroma to tumor cells, in which dense fibrosis develops because of reciprocal interactions between tumor cells and local dermal fibroblasts [37, 38]. CAFs are a major cell type in the complex multicell-type and immunosuppressive microcosmos, which creates a supportive tumor niche and constitutes a protective shell that blocks drug access and nurtures drug resistance against targeted therapies [4]. Unsurprisingly, CAFs are associated with poor prognosis in many cancer types [39–41]. One interesting and important characteristic of CAFs that has been described by *Calvo et al*. is the contribution of YAP1 as a master driver of the CAF phenotype [13]. Since *Calvo et al*. outlined the role of YAP1 in breast cancer-derived CAFs, nuclear YAP1 expression has been detected in CAFs of a broad spectrum of cancers, suggesting that its expression is one of their universal features [42–44]. It has become clear that for CAFs to have a tumor-supporting function, they require YAP1 [10, 45].

YAP1 is the main target of the Hippo pathway, whose role is to inactivate the nuclear localization and transcriptional coactivator activity of YAP1 [46]. However, it is unclear whether impaired Hippo signaling is a main promoter of nuclear YAP1 in CAFs because *Calvo et al*. did not find any evidence for decreased Hippo activity in this cell type. We previously reported that YAP1 is a part of the b-catenin signaling axis in CAFs that controls CAF behavior and demonstrated that loss of either of them is detrimental to CAF function [10, 16, 17]. In this study, two mouse models carrying either YAP1-deficient CAFs or YAP-overexpressing CAFs showed opposite effects on *in vivo* melanoma growth, reflecting the importance of YAP1 in CAFs and of CAFs for melanoma growth. Furthermore, we confirmed that YAP1 is essential for modulating cytoskeletal changes in CAFs and their ability to remodel the matrix. Coincidently, RNA-Seq revealed that YAP1 modulates CAF functions by controlling major sets of genes that are associated with cytoskeletal functions. The gene that stands out and is most downregulated after YAP1 loss is N-cadherin, a molecule that spearheads CAF-melanoma interactions and invasion after melanoma cells have undergone the switch from epithelial cadherin (E-cadherin) to neural cadherin (N-cadherin) [47]. N-cadherin is known to be expressed on CAFs and associated with the actin cytoskeleton through catenin molecules [48]. We observed that YAP1-deficient CAFs lose the ability to remodel the cytoskeleton and matrix and that N-cadherin is a downstream effector of YAP1 signaling, potentially suggesting that N-cadherin may indeed act as a YAP1-controlled molecular regulator of the actin cytoskeleton in CAFs.

Our results of YAP1 regulating N-cadherin have also been documented in other cell types. For example, suppressing YAP1 expression reduces N-cadherin expression in glioma cells [49]. One component of Hippo signaling, AJUBA, has a similar effect on N-cadherin as YAP1 [50]. However, it is unclear whether AJUBA requires YAP1 to activate N-cadherin. It was only shown that AJUBA can cooperate with the transcription factor Twist to activate the N-cadherin promoter. In addition to AJUBA/Twist, a plethora of other factors, including SET8 [51], FOXD1 [52], ZNF532 [52], CSNK2 [53], MZF1 [54], TRIM28 [55], activated Notch1 signaling [56], GSK3 inhibition [57], TGFb/TNF [58, 59], and SMAD4 [60], were shown to activate N-cadherin expression. These findings highlight the complex regulation of N-cadherin activity. In addition, YAP1 activity and N-cadherin expression can both be regulated by matrix stiffness [61].

N-cadherin is a member of the calcium-dependent adhesion molecule family of classical cadherins that mediates cell-cell adhesion and interaction [62]. The E- to N-cadherin switch in tumor cells is principally the switch from an invasion-suppressing cadherin [63] to a motility and invasion-promoting cadherin [64]. Accordingly, the E- to N-cadherin switch is negatively associated with melanoma patient survival as defined by overall low E-cadherin and high N-cadherin expression in patient specimens [65]. Melanocytes interact with keratinocytes and are retained in the epidermis since both express E-cadherin. When melanoma cells become invasive, the switch from E-cadherin to N-cadherin expression allows them to break away from keratinocytes, bind to N-cadherin-expressing fibroblasts, and invade the underlying dermis [66]. It was speculated that the release of melanocytes from keratinocyte control mediated by E-cadherin is a critical step in malignant transformation [67–72]. By switching from E-cadherin to N-cadherin, melanocytes not only are freed from keratinocyte control but also gain new capabilities and can interact with other N-cadherin-expressing cells, such as fibroblasts in the dermis. Many studies have implied an important role of cadherin switching in melanoma and dermal invasion [73]. The aberrant expression of N-cadherin in many types of solid tumors is critical for their aggressive phenotype, such as cancer cell invasion and tumor metastasis [74, 75]. Forced expression of N-cadherin in N-cadherin-negative melanocytes conferred elevated properties of migration to these nonmalignant cells [76]. Accordingly, reducing N-cadherin expression in human melanoma cell lines leads to decreased invasive capabilities [77]. Different mechanisms that control the E- to N-cadherin switch in tumor cells have been reported [47], including the PI3K/PTEN pathway that transcriptionally regulates the ‘cadherin switch’ through Twist and Snail [78]. However, it is unclear whether CAFs have any role in regulating this switch and influencing melanoma progression through the N-cadherin interaction.

Interestingly, we observed that the loss of N-cadherin expression in CAFs reduces N-cadherin in melanoma cells and their proliferative phenotype. N-cadherin, E-cadherin, and other adhesion receptors are continuously turned over at the membrane and contribute to the dynamic assembly and disassembly of adhesive junctions [79–81]. Upon internalization in response to different signals, cadherin molecules are either recycled or degraded. However, it remains unclear how the loss of N-cadherin in CAFs leads to the loss of N-cadherin in melanoma cells. One possible reason could be that the direct interaction between melanocytes and fibroblasts via N-cadherin is closely regulated. When CAFs lose N-cadherin molecules, homotypic N-cadherin interactions can no longer be maintained. Thus, melanoma cells initiate the degradation process to remove unneeded N-cadherin and downregulate N-cadherin expression. Another explanation could be that YAP1-deficient or N-cadherin-deficient CAFs are unable to produce cytokines or molecules that are necessary to maintain N-cadherin expression in melanoma cells. Potentially, ablating N-cadherin in CAFs could constitute a negative signal and reverse the E- and N-cadherin switch in melanoma cells since cadherin-mediated cell-cell adhesion needs to be maintained and is central to bridging neighboring cells and the cytoskeleton for sensing and responding to physical and mechanical changes in the stroma.

How changes in N-cadherin expression bring about these profound changes in cellular behavior in melanoma cells is partially understood. We now at least understand that N-cadherin is not only involved in cell-cell adhesion [33]. It has been demonstrated that N-cadherin may regulate PI3K-AKT signaling [76, 82]. Suppression of integrin-linked kinase (ILK) has a similar effect and reduces AKT signaling, N-cadherin expression, and melanoma cell migration and invasion [83]. However, whether the PI3K/PTEN/AKT signaling axis is more directly involved in N-cadherin expression was explored in detail by *Hao et al*., who showed that re-expression of PTEN in melanoma cells reverses the E- to N-cadherin switch, suppresses AKT signaling, and decreases the expression of EMT transcription factors of the TWIST and SNAI families [78]. In addition, while N-cadherin can be induced in melanoma cells in a SMAD4-dependent manner by TGFb treatment, this induction can be reversed by PI3K inhibition [78]. Cadherins can bind directly to PI3K [84]. However, in prostate cancer cells, only N-cadherin, but not E-cadherin, was able to activate PI3K/AKT signaling by recruiting PI3K to the N-cadherin complex [85]. N-cadherin can also influence AKT signaling in unorthodox ways by inhibiting isoform expression, e.g., in breast cancer cells, N-cadherin selectively suppresses AKT3 expression, and this suppression appeared to be the key to maintaining invasive behavior [86]. Whether this finding is relevant for melanoma is unknown, and further in-depth study is needed.

In addition to PI3K/AKT signaling, N-cadherin can also activate other signaling pathways, including Rac and Rho signaling. Similar to N-cadherin interacting with PI3K, it can also directly interact with several members of the Rho and Rac family of small GTPases. These interactions have been suggested to be an important aspect of cadherin-mediated cell-cell adhesion-activated signaling [87, 88]. Whether Rho/Rac or other pathways are key to the CAF-dependent N-cadherin signaling in melanoma cells outlined here is open for further research. Our data suggest that PI3K/AKT signaling is tightly linked to N-cadherin signaling in melanoma cells and that CAFs lacking N-cadherin can suppress N-cadherin expression in melanoma cells. This surprising finding highlights the importance of melanoma-stroma interactions and their largely untouched potential for novel therapeutic approaches [4].

Ablating or blocking N-cadherin in melanoma cells impairs their cell-cell adhesion, suppresses migration, and reduces their viability, suggesting that N-cadherin could be a therapeutic target in cancer treatment. Both peptides and monoclonal antibodies targeting N-cadherin have shown some efficacy in the preclinical setting. In an *in vivo* preclinical melanoma model, inhibition of N-cadherin function with a cyclic pentapeptide combined with chemotherapy led to enhanced tumor cell apoptosis and inhibition of tumor growth [89]. In another study, the activity of this cyclic pentapeptide N-cadherin inhibitor was determined *in vivo* to occur through altering AKT activity and altering vascular permeability. In a similar model for prostate cancer, the same drug mimicked the results of N-cadherin ablation in prostate cancer cells, reducing immunosuppression and enhancing tumor-infiltrating leukocyte-related therapy by modulating the expression of immune checkpoint inhibitors [90].

## Conclusion

In conclusion, we present novel insights into the reciprocal interaction between melanoma cells and CAFs and how their interactions promote melanoma cell proliferation and tumor progression. We demonstrate that YAP1 is a key activator of N-cadherin expression in CAFs and that the loss of N-cadherin in CAFs results in a loss of N-cadherin in melanoma cells. However, it remains to be further studied whether the E- to N-cadherin switch and EMT in melanoma cells could be reversed by targeting N-cadherin in CAFs and/or melanoma cells. In addition, the mechanism that controls the recycling, internalization, degradation, and expression of N-cadherin in melanoma cells by N-cadherin-negative CAFs needs to be further elucidated. Nevertheless, further exploration of YAP1 and N-cadherin as therapeutic targets to inhibit melanoma metastasis and improve targeted therapies and immunotherapies is warranted.

## Data Availability

All materials underlying this study are available from the corresponding author based on a material transfer agreement. RNA-Seq data were deposited in the GEO database (GSE208058).

## Abbreviations

α-SMA: Alpha smooth muscle actin
3D: Three dimensional
CAFs: Cancer-associated fibroblasts
CRM: Confocal reflection microscopy
ECM: Extracellular matrix
EMT: Epithelial-to-mesenchymal transition
DMEM: Dulbecco’s modified Eagle medium
FBS: Fetal bovine serum
GFP: Green fluorescent protein
KO: Knock out
LATS1/2: Large tumor suppressor kinases ½
MST1/2: Mammalian sterile twenty-like kinases 1/2 and
PBS: Phosphate buffered saline
PCR: Polymerase chain reaction
PI3K: The phosphoinositide 3-kinase
qPCR: Quantitative polymerase chain reaction
rtTA: reverse tet transactivator
RFP: Red fluorescent protein
siRNA: Small interfering RNAs
shRNA: Small hairpin RNAs
TNC: Tenascin C
TUNEL: Terminal deoxynucleotidyl transferase dUTP nick end labeling
YAP1: Yes-associated protein 1
